# Bilateral Testicular Epidermoid Cysts in a Man with Klinefelter Syndrome: A Case Report

**DOI:** 10.7759/cureus.11834

**Published:** 2020-12-01

**Authors:** Mya L Win, Devinder Kaur, Samson O Oyibo, Jeyanthy Rajkanna, Satyanarayana V Sagi

**Affiliations:** 1 Diabetes and Endocrinology, Peterborough City Hospital, Peterborough, GBR

**Keywords:** epidermoid cyst, testis, case report, testosterone hormone, klinefelter syndrome, testicular, hypergonadotropic hypogonadism, x chromosome, adult teratoma, multi-disciplinary teams

## Abstract

Klinefelter syndrome is a rare chromosomal disorder with at least one extra X chromosome in males resulting in male hypogonadism, androgen deficiency and impaired spermatogenesis. It is associated with an increased risk of certain malignancies; including leukemia, breast cancer, non-Hodgkin's lymphoma and mediastinal germ cell tumors, however, testicular tumors are rare in men with Klinefelter syndrome. Testicular epidermoid cysts are rare benign tumors affecting the testes. We report a case of bilateral testicular epidermoid cysts in a 30-year-old man known to have Klinefelter syndrome. He had an incidental finding of bilateral hard irregular-surfaced testes during routine assessment for testosterone replacement therapy. Biochemical investigation confirmed primary hypogonadism and ultrasound imaging demonstrated bilateral solid testicular masses with no blood flow seen within the lesions. The patient went on to have a right-sided radical orchiectomy with left-side sparing. The histology revealed features in keeping with that of a testicular epidermoid cyst with no evidence of malignancy. The patient was commenced on testosterone replacement therapy. This case emphasizes the importance of routine physical examination of the male external and internal genitalia when considering testosterone replacement therapy.

## Introduction

Klinefelter syndrome is a state of hypergonadotropic hypogonadism in male individuals due to having at least one extra X chromosome. The 47, XXY karyotype is the commonest human sex chromosome disorder having a prevalence of one in 500-600 males [1]. Klinefelter syndrome is characterized by small testes, oligospermia or azoospermia, gynecomastia, sparse body hair, hyalinization and fibrosis of the seminiferous tubules, low testosterone levels and raised gonadotropin levels [2]. Klinefelter syndrome is associated with an increased risk of malignant diseases including leukemia, breast cancer, non-Hodgkin’s lymphoma and mediastinal germ cell tumors. Despite this studies have not found an increased frequency of testicular cancer in men with Klinefelter syndrome [3,4].

Testicular epidermoid cysts (TEC) are benign tumors and although the most common benign tumors of the testes, they account for only 1-2% of all testicular tumors. They usually measure 1-3 cm in size and affect men in their second to fourth decade of life [5,6]. Most cases are incidental findings during routine physical examination while some have been reported by the patients after ignoring a testicular nodule for several years. Physical examination usually reveals a discrete, hard nodule within the testis. Depending on tumor size and further investigations, testicular sparing surgical resection is usually adequate treatment [5,6]. TEC have been reclassified as "teratoma of prepubertal type" within the group of germ cell tumors unrelated to germ cell neoplasia in situ. The International Classification of Disease for Oncology code (ICD-O code 9084/0) attributed to TEC denotes the benign behavior of these types of tumors [7].

Testicular tumors occurring in patients with Klinefelter syndrome are rare. Bilateral TEC occurring in patients with Klinefelter syndrome are even rarer. We present a case of bilateral TEC occurring in a young man with Klinefelter syndrome.

## Case presentation

Medical history and demographics

A 30-year-old man was referred to the endocrine outpatient clinic to initiate testosterone replacement in view of worsening symptoms of tiredness, lethargy and erectile dysfunction. He was diagnosed with Klinefelter syndrome during childhood but had not had any testosterone replacement for up to 10 years due to poor adherence to treatment. He had a medical history of mild asthma. He did not have cryptorchidism in childhood. He was a smoker and occasional drinker. He had not fathered a child before. He was not married but had a partner and both expressed their desire to have children in the future. He had no family history of testicular pathology.

On physical examination, he had gynecomastia and sparse body hair over his arms and legs. However, he had normal pubic hair and normal penile size. His both testes were very hard with irregular surfaces: the right testes measuring 25 ml in size and the left testes measuring 8-10 ml in size. He weighed 83.6 kg with a height of 1.93 m (BMI 22.4).

Investigations

His laboratory tests showed very low serum testosterone levels, elevated luteinizing hormone and follicle stimulating hormone levels, which were consistent with primary gonadal failure (hypergonadotropic hypogonadism). Testicular tumor markers such as alpha-fetoprotein and serum beta human chorionic gonadotropin (beta HCG) were normal. Thyroid function test, renal function test, liver function test, prostate specific antigen, vitamin D, calcium and prolactin levels were all within normal limits (Table 1).

**Table 1:** Baseline blood results for patient with laboratory reference ranges

Blood test	Normal reference range	Patient’s results
Testosterone	10-38 nmol/L	5.7
Prolactin	<330 mU/L	275
Luteinizing hormone	2-9 U/L	21
Follicle-stimulating hormone	2-13 U/L	50
Thyroid-stimulating hormone	0.3-4.2 mU/L	1.37
25-hydroxy vitamin D	>50nmol/L	81.2
Alpha-fetoprotein	<7 ng/ml	2
Beta Human Chorionic Gonadotropin	0-4 IU/L	2
Prostate specific antigen	<2.5 mg/L	0.65
Haemoglobin	130-180 g/L	152
Haematocrit	0.400-0.530	0.428

**Figure 1:**
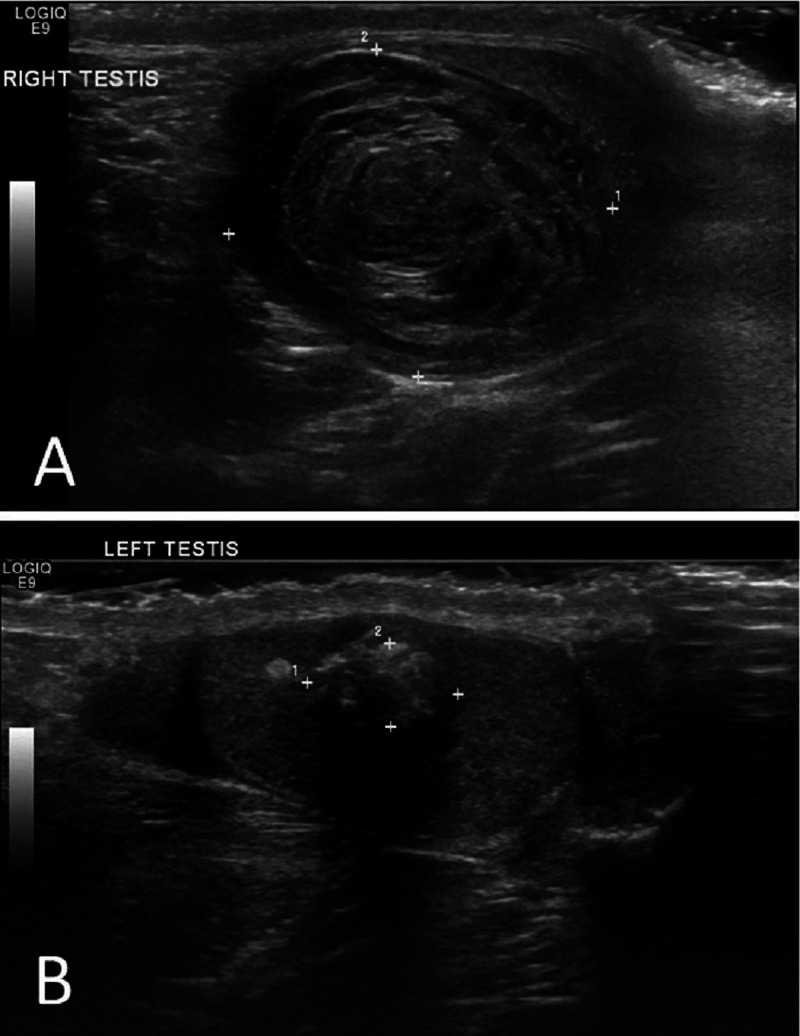
Ultrasound scan demonstrating bilateral testicular epidermoid cysts. (A) Right testicular cyst demonstrating the characteristic “onion ring” appearance. (B) Left testicular cyst demonstrating a mixed/alternating echogenic pattern.

**Figure 2:**
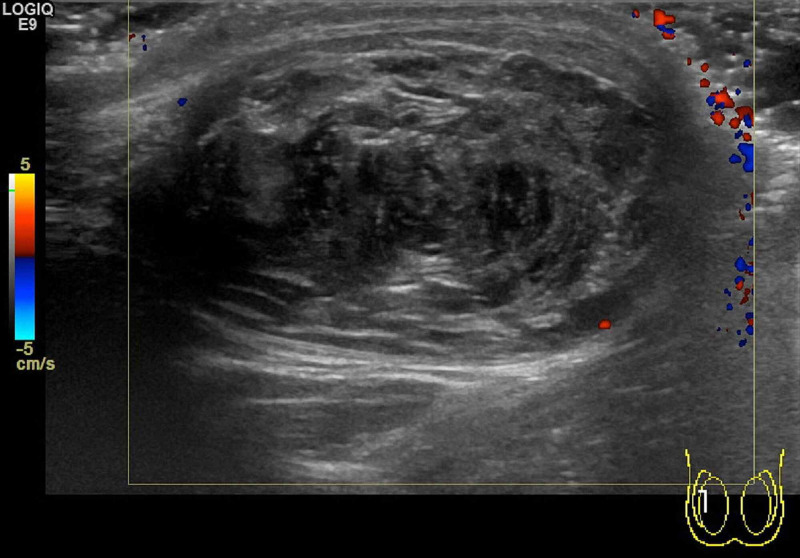
Doppler study of right testicular cyst demonstrating absence of vascular flow.

An ultrasound scan of his testes demonstrated bilateral solid testicular masses with no blood flow seen within the lesions. There was very little identifiable normal testicular tissue seen within the right testis, which was replaced by a large mass measuring 3.4 cm x 2.7 cm x 2.3 cm. The left testis contained an echogenic, well defined mass measuring 0.9 cm x 0.65 cm x 0.5 cm (Figure 1 and Figure 2).

A bone density scan revealed slightly low bone mineral density (BMD) in the spine (T-score -1.9, BMD 0.877 g/cm^2^) but normal in the hip (T-score -1.0, BMD 0.878 g/cm^2^). A computerized tomography (CT) scan of his chest, abdomen and pelvis revealed no abnormalities.

**Figure 3:**
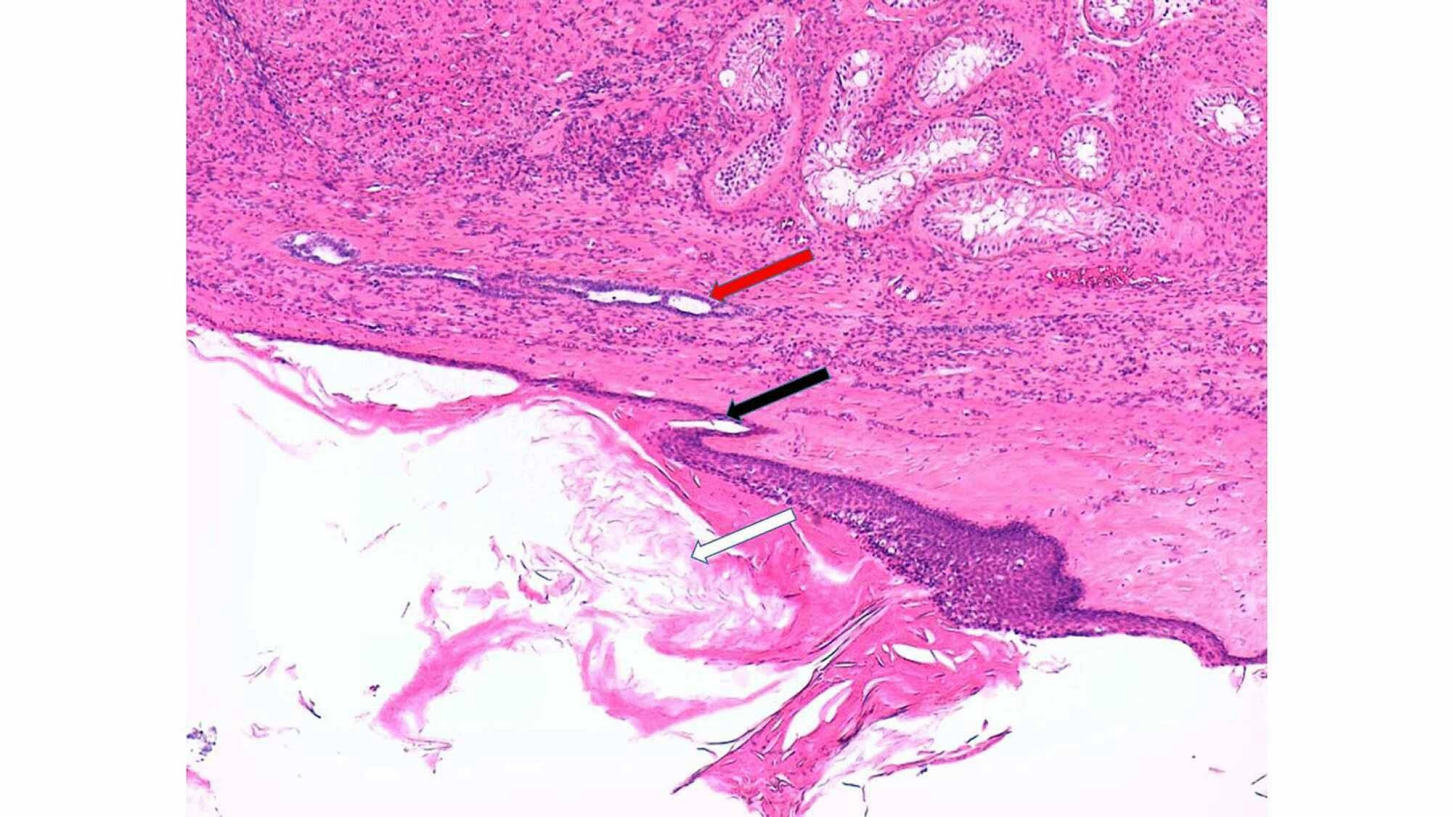
Histological section of the right epidermoid cyst (haematoxylin & eosin x50) demonstrating compressed normal testicular parenchyma (red arrow), stratified squamous epithelial cell with a granular layer (black arrow), and cyst lumen filled with keratin debris (white arrow).

The histology after right orchiectomy revealed a 3.1 cm x 2.2 cm x 2.0 cm cystic lesion that contained lamellated keratin and was lined by squamous epithelium with a granular layer. The features were consistent with an epidermoid cyst. There was no evidence of intra-tubular germ cell neoplasia or malignancy (Figure 3).

Treatment

After a Multidisciplinary Team assessment, the patient went on to have a right-sided orchiectomy (two months after initial examination) as opposed to enucleation because of the size of the lesion and the fact that malignant changes could not be confidently ruled out. After receiving the histology report a decision was made to spare the left testis and keep the left testicular mass under ultrasound surveillance. Sperm retrieval and cryopreservation was discussed with the patient. His post-op serum testosterone level had fallen to 2.7 nmol/L. Because the patient had not been on testosterone replacement for a long time and had become very symptomatic, he was not keen on waiting to see if he would get funding approval for sperm retrieval and cryopreservation for future assisted fertility treatment. He also understood that the chances of success would be low. The patient was then put on testosterone replacement therapy in the form of a topical gel (40-60 mg) to apply daily.

Outcome and follow-up 

The patient remained well and was being followed up with regular ultrasound scanning of the spared left testis. The appearance of the left-sided epidermoid cyst remained unchanged over five years of ultrasound surveillance.

## Discussion

TEC are benign tumors classified under "teratoma of prepubertal type". They are the most common benign tumors of the testes, but account for only 1-2% of all testicular tumors and commonly affect men in their second to fourth decade of life [5,6]. However and of high importance, malignant germ cell tumors of the testes are 50 times more common than TEC so malignant germ cell tumors must always be high on the differential diagnoses list for young men presenting with a testicular mass. Affected individuals require full investigation to rule out malignancy [5,6]. Tumor markers (beta HCG, lactate dehydrogenase, alpha-fetoprotein) are negative in patients with TEC. Ultrasound scan demonstrates a unilocular cystic mass with a hyperechoic rim. Within the lesion there are alternating hypoechoic and hyperechoic concentric rings representing layers of compacted keratin and desquamated squamous cells, giving it a characteristic “onion ring” appearance (this "onion ring" appearance is found in about 60% of cases). Others give a bull’s eye appearance consisting of a hyperechoic capsule, hypoechoic content of fat and water, and a hyperechoic centre of keratin debris. An avascular mass indicating no blood flow is seen on colour Doppler flow study [5,6]. Price criteria are used for the histological diagnosis of an epidermoid cyst: (i) the cyst located within the testis parenchyma; (ii) cyst lumen contains keratin; (iii) cyst wall comprising fibrous tissue with complete or incomplete squamous epithelial inner lining; (iv) the cyst must not contain any teratomatous components (e.g., sebaceous glands, hair); and (v) no scar may be seen in the remaining testicular parenchyma [5].

The biochemical and ultrasound findings are usually enough for a confident pre-operative diagnosis, such that testis-sparing surgery (e.g., enucleation or wedge resection) can be performed, with concomitant use of intra-operative frozen section histologic analysis, to rule out occult malignancy. However, total orchiectomy must be done if in doubt [6]. Testicular sperm extraction (TESE) can be carried out before surgery and testosterone replacement therapy so as to preserve sperm for future assisted fertility techniques [8].

A large cohort study demonstrated that men with Klinefelter syndrome may be at increased risk for developing lung cancer, non-Hodgkin lymphoma, breast cancer and mediastinal germ cell tumors (extra-gonadal tumors). The incidence of testicular cancer was not high and there were no cases of mortality from testicular cancer found in this study [3]. The combination of underdeveloped and undescended testes that occurs in patients with Klinefelter syndrome would theoretically render them at high risk for testicular neoplasia. However, another large study revealed that the incidence of testicular cancer reported in patients with Klinefelter syndrome was not high [9].

Up until 2018 there had been only eight cases of TEC found in individuals with Klinefelter syndrome reported in the literature. Of these cases only one had bilateral involvement. Their ages ranged from six to 38 years and all cases underwent orchiectomy [10]. Reasons for orchiectomy being performed instead of enucleation were mainly that teratomatous or malignant changes could not confidently be ruled out. As a consequence of the paucity of cases there is no evidence-based data concerning orchiectomy versus enucleation for TEC in patients with Klinefelter syndrome. Many patients with Klinefelter syndrome have very small testes and the presence of a large TEC can obliterate any functioning testicular parenchyma. This case emphasizes the need to include all patients in the center of such surgical decisions, especially concerning sperm retrieval, prosthesis and psychological well-being.

The patient in our case report had a lesion that satisfied both the ultrasonic and histological criteria for the diagnosis of a TEC. However, as there was very little testicular tissue detectable and any malignant changes could not be ruled out with 100% confidence on ultrasound, an orchiectomy was deemed safer. The fact that the left testicular mass remains the same size on yearly ultrasound scanning over five years confirms it is a similar but smaller epidermoid cyst.

The patient in this case report did not complain of any testicular mass or discomfort. The testicular masses were coincidental findings during examination of the male genitalia before testosterone replacement therapy. This patient had right-sided orchiectomy as opposed to enucleation because malignancy could not be confidently ruled out as in the previous case reports. The left-sided testicular mass was left alone after the right-sided mass was confirmed benign. Sparing the left side also helped the psychological well-being of the patient.

## Conclusions

We have presented a rare case of bilateral testicular epidermoid cysts occurring in a young man with Klinefelter syndrome. This case was found by chance during routine examination before initiation of testosterone replacement therapy. This case emphasizes the importance of routine physical examination of the male external and internal genitalia when considering testosterone replacement therapy. We hope that this case report not only adds to the existing literature but also contributes to gaining insight into this rare benign tumor of the testis.
